# Preferential Tax Policies: An Invisible Hand behind Preparedness for Public Health Emergencies

**DOI:** 10.34172/ijhpm.2020.139

**Published:** 2020-08-01

**Authors:** Yong Fan, Shujuan Yang, Peng Jia

**Affiliations:** ^1^School of Public Finance and Tax, Central University of Finance and Economics, Beijing, China.; ^2^International Institute of Spatial Lifecourse Epidemiology (ISLE), Hong Kong, China.; ^3^West China School of Public Health and West China Fourth Hospital, Sichuan University, Chengdu, China.; ^4^Institute for Disaster Management and Reconstruction, Sichuan University, Chengdu, China.; ^5^Department of Land Surveying and Geo-Informatics, The Hong Kong Polytechnic University, Hong Kong, China.

**Keywords:** COVID-19, Tax, Public Finance, Epidemic, Emergency, China

## Abstract

The control and prevention of public health emergencies can face severe challenges, especially financial and material challenges during the coronavirus disease 2019 (COVID-19). Enabling and ensuring smooth financial and material flows across levels, within the country, and across countries are essentially important to preparedness for global health emergencies, which cannot easily be achieved without being facilitated by preferential tax policies. China’s preferential tax policy practice developed at early stages of the COVID-19 pandemic could be useful experiences which can be adapted to unique contexts of other countries, so different stakeholders including citizens could be effectively motivated and involved in the fight against the COVID-19 pandemic. However, we should see that these policies are temporary and issued as an afterthought. There is still much to learn about how epidemic responders and policy-makers can make the most of each other’s expertise to fit into the wider information architecture of epidemic response.

 The control and prevention of public health emergencies can face severe challenges, especially social and economic challenges, which, according to what has been happening in different countries during the coronavirus disease 2019 (COVID-19), could be not necessarily related to socioeconomic status of the country.^1,2^ For example, the emergency and disaster response system of the US is designed to be bottom-up, where responses are intended to begin at the local level with state and federal governments stepping in to assist as needed.^3^ In such societies, a recent study during the COVID-19 showed that, while many local governments were fiscally stressed prior to the COVID-19 outbreak, the drop in sales tax revenue due to the imposition of “stay-at- home” or “shelter-in-place” orders has severely threatened both their ability to continue to respond to the COVID-19 and their ability to remain solvent.^4^ The national support cannot fairly benefit or reach every single hospital, due to either social or geographical inequalities, or just national-level slow response. Even the global support may not be able to reach every country equally or timely, due to, for example, the sudden shift of urgent need for COVID-19 prevention materials from eastern to western countries during February-March 2020. This has left many vulnerable regions or communities disproportionately suffered from the local pandemic. Enabling and ensuring smooth financial and material flows across levels (eg, donation from citizens to hospitals), within the country (eg, transport of goods), and across countries (eg, importation) are essentially important to preparedness for public health emergencies. This may be temporarily realized by some short-term stimulus measures that could help individuals and businesses cope with the ravages of the COVID-19 and produce short-term results at best.^5^ However, long-term goals including economic recovery cannot easily be realized without being facilitated by an invisible hand behind – preferential tax policies.

 China’s preferential tax policy practice developed at early stages of the COVID-19 pandemic could be useful experiences which can be adapted to unique contexts of other countries, so different stakeholders including citizens could be effectively motivated and involved in the fight against the COVID-19 pandemic. In February 2020, the Chinese government issued a series of preferential tax policies in relevant areas and industries to strengthen COVID-19 prevention and control, which have gone in effect since January 1, 2020 (Figure).^6^

**Figure:**
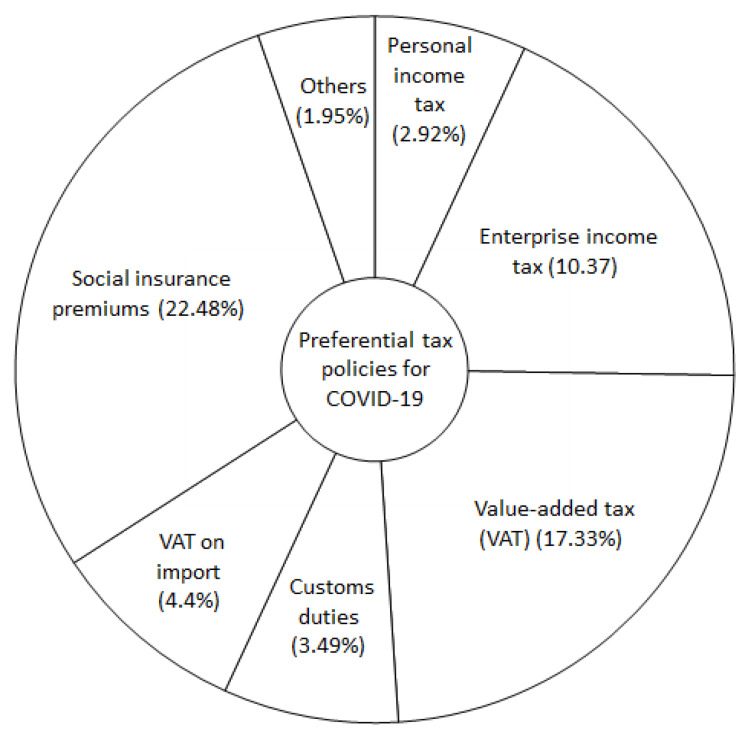
Percentages in the Central Government’s Revenue in 2019 of Seven Categories of Preferential Tax Policies Issued by the Chinese Government for COVID-19 Control and Prevention. Abbreviations: COVID-19, coronavirus disease 2019; VAT, value-added tax.

 They include exempting personal income tax of temporary subsidies and bonuses of health professionals for their work related to COVID-19 control and prevention; increasing the refund and deduction of the enterprise income tax and value-added tax (VAT) of enterprises producing and transporting COVID-19 prevention materials; removing customs duties for imported COVID-19 prevention materials; increasing tax deduction for expenditures of COVID-19-related personal and enterprise donation to governments, public charitable organizations, or directly to hospitals; alleviating the income tax and VAT of enterprises seriously affected by COVID-19; and reducing or exempting the employers’ and employees’ contribution to pension, unemployment, and work injury insurance premiums (Table).

**Table:** Detailed Preferential Tax Policies Issued by the Chinese Government for COVID-19 Control and Prevention

**Tax**	**Policies**
Personal income tax	Personal income tax is exempted for temporary work subsidies and bonuses for healthcare workers for COVID-19 prevention and control work.
	Personal income tax is exempted for medical supplies for COVID-19 prevention that individuals obtain from employers.
	Full deduction before personal income tax is allowed based on COVID-19 prevention goods donated through public charitable organizations or governmental agencies.
	Full deduction before personal income tax is allowed based on COVID-19 prevention goods donated directly to hospitals.
Enterprise income tax	The maximum carry-over period of the loss occurring in 2020 for enterprises severely affected by COVID-19 can be extended up to eight years.
	A one-time deduction before enterprise income tax is allowed for enterprises that expand production capacity and purchase equipment for producing COVID-19 prevention goods.
	Full deduction before enterprise income tax is allowed based on COVID-19 prevention goods donated through public charitable organizations or governmental agencies.
	Full deduction before enterprise income tax is allowed based on COVID-19 prevention goods donated directly to hospitals.
VAT	VAT incremental allowance tax is fully refunded for enterprises that produce COVID-19 prevention goods.
	VAT is exempted for enterprises that transport COVID-19 prevention goods.
	VAT is exempted for enterprises that provide public transport services, living services, and express delivery services of residents’ essential living materials.
	VAT is exempted for gratis donation of goods for COVID-19 control and prevention.
	VAT is temporarily reduced or exempted for small-scale taxpayers.
Customs duties	Customs duties are exempted for COVID-19 control and prevention goods imported by health sectors.
	Customs duties are exempted for imported COVID-19 control and prevention goods donated.
VAT on import	VAT on import is exempted for imported COVID-19 control and prevention goods donated.
Social insurance premiums	Employer’s contribution to pension, unemployment, and work injury insurance is temporarily reduced or exempted.
	Employee’s contribution to pension, unemployment, and work injury insurance is temporarily reduced or exempted.
	Employer’s contribution to basic medical insurance is temporarily reduced or exempted.
Others	Consumption tax, urban maintenance and construction tax, educational surtax, and local education surtax are exempted for gratis donation of goods for COVID-19 control and prevention.
	Reducing or exempted urban land use tax is encouraged to support renters to reduce property rents for individually-owned businesses.

Abbreviations: VAT, value-added tax; COVID-19, coronavirus disease 2019.

 The issuance of these preferential tax policies, benefiting many aspects of public health affected by COVID-19, is a timely tax policy response. These policies are not only an encouragement for individuals to be involved, but also an incentive for enterprises to participate in COVID-19 control and prevention through practical actions, such as producing more prevention materials or providing more services. In fact, what has happened, motivated by these policies, is as expected: more and more healthcare professionals have been willing to participate in front-line prevention work because they are not only encouraged in spirit, but also well treated in financial compensation; also, the situation of having insufficient supplies of prevention materials in China has been quickly reversed and gradually returned to normal. Stimulated by both market demand and temporary tax policies, the production costs of products have been reduced, which made many enterprises attempt to expand their production capacity or add new production lines to meet the needs of domestic markets.

 The tax policy measures taken so far in China have demonstrated a high premium placed on COVID-19 prevention and control in the agenda of the central government. They have benefited not only China, but the whole world, especially after the COVID-19 epidemic became a pandemic (officially declared on March 11, 2020). For example, some enterprises under such tax policy motivation have produced more COVID-19 prevention materials and goods, which are being exported to other countries in the fight against COVID-19: nearly 700 000 mouth masks from China arrived in the Netherlands on March 21, 2020, which is the first shipment of a multimillion-dollar order that will arrive in the Netherlands in the coming weeks; a total of 5 million COVID-19 rapid test kits were purchased from China and will be delivered to Brazil by middle April; 145 life-support machines and emergency ventilators were sent to Serbia on March 25, 2020, which is just one of many purchasing orders from 35 countries. These goods have been distributed as quickly as possible to the places where the need was the greatest, which have played a critical role in their fight against COVID-19 before local enterprises can meet their demand at a much less extortionate price.

 Although such tax policy responses reflect the flexibility of China’s tax policies when dealing with public health emergencies and well reserved tax policies, we should also see that these policies are temporary and issued as an afterthought to respond to the COVID-19 epidemic that has already occurred. They are not stably embedded in the current tax law system in China, which otherwise would have comprehensively strengthened the public health capacity of the country. There is still much to learn about how epidemic responders and policy-makers can make the most of each other’s expertise and how preferential fiscal and tax policies can fit into the wider information architecture of epidemic response. In the future, with the increase of environmental and public health risks,^7^ all countries should think about questions such as: can such tax preferential policies be integrated into the national basic tax system, making them an integral part of the national system of public health, especially in disease control and prevention?^8^ Can they play a role in alleviating adverse (side) effects of public health emergency events? Only combining financial policies and public health governance in an efficient way can make it possible to enable a larger supportive role to be played by financial policies in public health governance. Also, such considerations can provide financial and tax assistance not only to those enterprises and their employees that are affected by emergency events, but also to those directly suffering as a result of emergency events from a humanitarian perspective, such as health professionals working in hospitals and in the field for epidemic control, patients and their families.

 China’s public finance practice in the fight against the COVID-19 pandemic could be useful and adapted to other countries to incentivize corporations and individuals to participate in COVID-19 control and prevention under their respective cultures and institutions. Some efforts are also observed in other countries, such as tax relaxation in both developed (eg, the United States^9^) and developing countries (eg, Indonesia^10^). Special and urgent attention should be given to both low- and middle-income countries whose fragile fiscal and health systems and relatively lower tax morale may be challenged by the forthcoming pandemic,^11,12^ and also to high-income countries whose existing high tax rates have left citizens less capacity to support public health emergencies without additional preferential policies; for example, a call for lawmakers to avoid arbitrarily limiting tax provisions and raising taxes during this time has been made in the United States.^9^ Some challenges should be foreseen when implementing such measures in other countries with different public health emergency and disaster response systems. For example, it may be difficult to implement financial incentive policies or measures consistently across the US, due to the bottom-up design of their emergency and disaster response system^4^; the effects of doing so would be more heterogeneous due to the varying financial positions before the COVID-19 outbreak.^13^

 Global collaboration in reducing and even exempting customs duties of epidemic prevention materials in all countries could extraordinarily facilitate the balance between supply and demand of those goods, also helping trigger and accelerate development and innovation of epidemic prevention equipment.^14^ However, Rome was not built in a day. We need to step up our efforts soon in the fight against future epidemics.^7^ The pandemic is a double-edged sword: it hurts communities and humans while speeding up building a community of human destiny.^15^ “Only a global victory can end this pandemic, not a temporary rich countries’ win.”

## Acknowledgement

 We thank the National Natural Science Foundation of China (“Research on Tax Reform,Tax Administration and Enterprise Tax Compliance,” Grant No. 71973159) and the International Institute of Spatial Lifecourse Epidemiology (ISLE) for the research support.

## Ethical issues

 Not applicable.

## Competing interests

 Authors declare that they have no competing interests.

## Authors’ contributions

 PJ conceptualized and designed the study. All authors collected the data, wrote and revised the initial manuscript, and approved the final manuscript.

## Authors’ affiliations

^1^School of Public Finance and Tax, Central University of Finance and Economics, Beijing, China. ^2^International Institute of Spatial Lifecourse Epidemiology (ISLE), Hong Kong, China. ^3^West China School of Public Health and West China Fourth Hospital, Sichuan University, Chengdu, China. ^4^Institute for Disaster Management and Reconstruction, Sichuan University, Chengdu, China. ^5^Department of Land Surveying and Geo-Informatics, The Hong Kong Polytechnic University, Hong Kong, China.

## References

[1] Lee VJ, Ho M, Kai CW, Aguilera X, Heymann D, Wilder-Smith A (2020). Epidemic preparedness in urban settings: new challenges and opportunities. Lancet Infect Dis.

[2] Newmark Z. Italian Politicians Criticize Netherlands Over Lack of Solidarity. NL Times. March 31, 2020. https://nltimes.nl/2020/03/31/italian-politicians-criticize-netherlands-lack-solidarity.

[3] McDonald BD, Goodman CB, Hatch ME. Tensions in State-Local Intergovernmental Response to Emergencies: The Case of COVID-19. OSF Preprints. 2020. 10.31219/osf.io/cnzt6

[4] McDonald BD, Larson SE. Implications of the Coronavirus on Sales Tax Revenue and Local Government Fiscal Health. SSRN Electronic Journal. 2020. 10.2139/ssrn.3571827

[5] McCoskey M, Narotzki D. The COVID-19 Economy: Now is the Time to Focus on Long-Term Recovery. Tax Notes Federal; 2020. https://ssrn.com/abstract=3638156.

[6] State Taxation Administration. Guidance for Preferential Tax Policies for COVID-19 Control and Prevention. State Taxation Administration; 2020.

[7] Jia P, Yang S (2020). Are we ready for a new era of high-impact and high-frequency epidemics?. Nature.

[8] Jia P, Yang S (2020). China needs a national intelligent syndromic surveillance system. Nat Medi.

[9] Pomerleau K. Tax Policy and the Federal Response to COVID-19. American Enterprise Institute (AEI); 2020.

[10] Munandar MH (2020). Analysis the effectiveness of tax relaxation due to Covid-19 pandemy on Indonesian economic defense. Lex Scientia Law Review.

[11] Nkengasong JN, Mankoula W (2020). Looming threat of COVID-19 infection in Africa: act collectively, and fast. Lancet.

[12] Cano MC. COVID-19 will Reshape African Tax Landscape. International Tax Review; 2020.

[13] Larson SE, McDonald BB. When the Beaches Close: Impact of COVID-19 upon County Fiscal Health in Florida. SSRN Electronic Journal. 2020. 10.2139/ssrn.3594898

[14] Fan Y. Customs duties of these goods will be reduced since 2020. In: Lu Y, editor. The State Council of China; 2020.

[15] Jia P, Wang Y (2019). Global health efforts and opportunities related to the Belt and Road Initiative. Lancet Glob Health.

